# Post-endoscopy esophageal adenocarcinoma and root cause analysis in Auckland, New Zealand

**DOI:** 10.1055/a-2676-3883

**Published:** 2025-09-30

**Authors:** Seong Shin, Dongyeon Kang, Russell S Walmsley

**Affiliations:** 1 1406 Gastroenterology, Te Whatu Ora Health New Zealand Waitemata, Auckland, New Zealand; 2 1403 Gastroenterology, North Shore Hospital, Auckland, New Zealand; 3 62710 Faculty of Medical and Health Sciences, The University of Auckland, Auckland, New Zealand

**Keywords:** Endoscopy Upper GI Tract, Barrett's and adenocarcinoma, Precancerous conditions & cancerous lesions (displasia and cancer) stomach, Malignant strictures

## Abstract

**Background and study aims:**

Post-endoscopy esophageal adenocarcinomas (PEEC) challenge timely diagnosis of esophageal adenocarcinomas (OAs). This study aimed to determine prevalence of PEECs in Auckland region and elucidate the most plausible causes through a root-cause analysis framework.

**Patients and methods:**

OA cases diagnosed in Auckland from 2013 to 2022 were retrieved from the New Zealand Cancer Registry (NZCR). Electronic clinical data were collected via the Regional Clinical Portal software. The primary outcome was PEEC prevalence, defined as OA diagnosed 6 to 36 months following an esophagogastroduodenoscopy (EGD) that failed to detect the cancer. Identified PEECs were classified into six categories.

**Results:**

Among 633 OA cases, 45 (7.1%) were PEECs. A higher prevalence of PEEC was observed in patients with Barrett’s esophagus (BE) (18.1% vs 2.7%), undergoing surveillance EGDs (52.6% vs 3.6%) and with early-stage cancers. Root-cause analysis delineated the PEEC causes, classified as follows: A (17.8%): lesion was identified, endoscopic assessment was adequate, follow-up was appropriately planned and executed, yet PEEC developed; B (17.8%): follow-up was delayed due to administrative factors; C (22.2%): follow-up decisions were inappropriate; D (22.2%): inadequate endoscopic assessment; E (11.1%): lesion was unidentified despite adequate assessment; and F (8.9%): lesion was unidentified and assessment was inadequate. Categories B, C, D, and F comprised 71.1% of cases deemed potentially avoidable.

**Conclusions:**

Auckland’s PEEC prevalence aligns with international post-endoscopy upper gastrointestinal cancer rates. Root-cause analysis underscores that a significant proportion of PEECs may be preventable with improved clinical practice.

## Introduction

Esophageal cancer represents a major health burden globally, being the eighth most prevalent cancer and responsible for 3.8% of all cancer cases and 5.4% of cancer deaths 1 . In 2017, New Zealand reported an incidence rate of 3.7 per 100,000 individuals 2 . The 5-year survival rate for esophageal cancer remains below 20% in Western countries 3 , yet early-stage esophageal cancer, managed promptly through surgical or endoscopic resection, can yield significantly higher 5-year survival rates, ranging between 75% and 90% 4 .

Esophagogastroduodenoscopy (EGD) with biopsy is the gold standard for diagnosing upper gastrointestinal cancer (UGIC) 5 ^.^ However, a proportion of UGICs are diagnosed within a short time frame of an endoscopy that did not identify the malignancy. These post-endoscopy upper gastrointestinal cancers (PEUGICs) have been defined as UGICs diagnosed in patients who had an EGD that did not identify the cancer within 6 to 36 months prior to diagnosis 6 7 . Several studies have highlighted the prevalence of PEUGICs 6 7 8 9 10 11 12 . In a meta-analysis of 25 heterogenous studies, 7926 of 81,184 included UGICs (9.8%) were considered PEUGICs 13 . Characteristics associated with PEUGICs included a less advanced cancer stage, younger age, female gender, and reduced likelihood of presenting with alarm symptoms 13 . A root-cause analysis of PEUGICs has been undertaken by Kamran et al, who examined PEUGICs across 1327 patients with UGICs identified at two United Kingdom endoscopy providers and highlighted that the most prevalent causes for PEUGICs were suboptimal evaluation of premalignant conditions, insufficient endoscopic quality, and poor surveillance planning 7 .

Esophageal adenocarcinoma (EA) is a subset of UGICs that develop from dysplastic changes in metaplastic epithelium of the esophagus over several years. Intestinal metaplasia of the esophagus, also known as Barrett’s esophagus (BE), occurs in 1% to 2% of the western population and has an annual progression rate to high-grade dysplasia (HGD) or EA of approximately 0.3% to 0.8% 14 15 . Surveillance protocols have been developed for patients with BE for early detection of dysplastic changes and EAs. However, there is a growing body of evidence describing occurrence of EAs before the next recommended endoscopic examination following an EGD that were deemed negative for HGD or EA 16 . A population-based cohort study performed across three Nordic countries revealed that 25% to 46% of esophageal EAs were diagnosed within 50–365 days from an index EGD that diagnosed non-dysplastic BE 17 . The American Gastroenterological Association introduced the term post-endoscopy EA (PEEC) to describe these PEUGICs that were EAs and to address the issue of BE endoscopy quality 16 17 18 . Our study aimed to determine the proportion of EAs that were PEECs in the Auckland metropolitan area and to perform a systematic root-cause analysis to elucidate the most plausible explanations.

## Patients and methods

### Study design and population

**Fig. 1:**
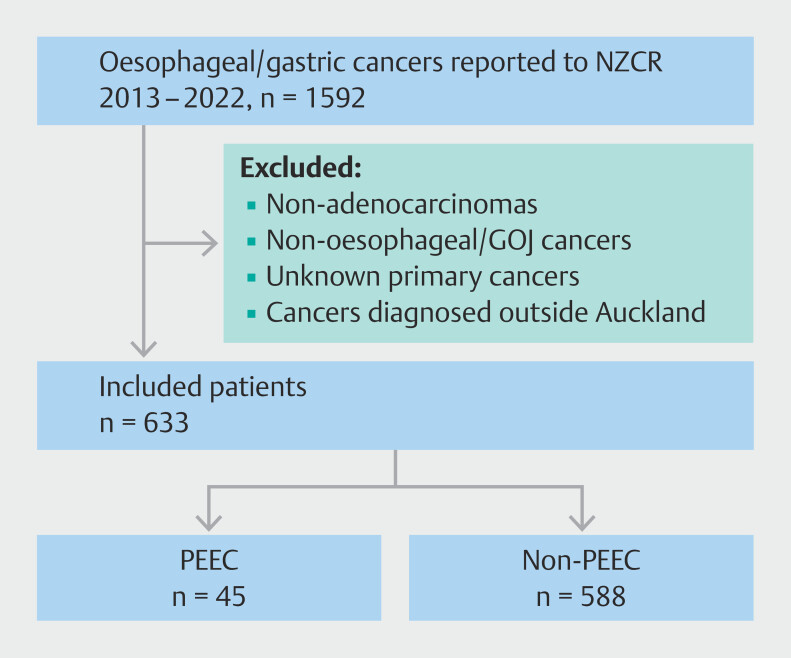
Flowchart of eligible patients identified from NZCR database and exclusion criteria.

This retrospective, observational study was conducted on a population of 1.6 million individuals, served by Te Whatu Ora Te Toka Tumai, Counties Manukau and Waitematā. The cohort consisted of patients diagnosed with EA between 2013 and 2022, as recorded by the New Zealand Cancer Registry (NZCR). Date of cancer diagnosis was assumed to be the date of cancer-diagnosing histology report. Inclusion criteria were adults aged > 18 years with esophageal or gastroesophageal junction (GOJ) cancer, classified under the International Classification of Diseases 10 ^th^ revision, as a malignant neoplasm of the esophagus or stomach (codes C15 and C16 respectively). GOJ cancer was included in this study of PEECs even though it is classified as a neoplasm of the stomach by the NZCR, because it is a common location where adenocarcinomas arise and it is also a site where an adequate endoscopic examination is more difficult and could pose a potential area for improvement. Exclusion criteria included patients with malignancies outside the esophagus or GOJ, non-adenocarcinoma histology, or those first diagnosed outside Auckland ( Fig. 1 ).

### Data acquisition

Clinical data were obtained from electronic health records via the Regional Clinical Portal software (Orion Health, Auckland, New Zealand). These records included endoscopy, histology, and radiology reports as well as multidisciplinary meeting correspondence, hospital discharge summaries, and clinic letters. Previous EGDs were verified using the Provation MD system (Minneapolis, Minnesota, United States). In addition, data from private endoscopy procedures were procured through direct communication with the clinical directors of the respective private entities. Data collection was conducted by two researchers with full access to identifiable patient information, under the oversight of a third researcher without direct data access.

### Outcome measures

The primary outcome measure was prevalence of PEEC, defined as EAs diagnosed 6 to 36 months following an index EGD that did not identify the EA. Patients who had an EGD within 6 months of cancer diagnosis were excluded because these EGDs could have been part of diagnostic workup. In cases where multiple EGDs were performed within the 6- to 36-month window before diagnosis, the EGD closest to the cancer diagnosis date was designated as the index EGD.

Data collection encompassed: 1) patient variables including gender, age, ethnicity, and smoking status; 2) details of endoscopic examinations: location where EGD was performed; indication and acuity of procedure; patient tolerance; number of biopsies taken; photo-documentation and description of key landmarks and premalignant conditions such as BE. If EGD reports were unavailable, it was presumed that the procedure was conducted in an outpatient setting by a senior medical officer employing an adequate technique. For BE patients, the number of biopsies taken was evaluated against BE segment length to assess adherence to the modified Seattle biopsy protocol 19 20 21 ; 3) endoscopist level of training; 4) oncological data such as cancer stage and its location. When discrepancies arose from different sources, the multidisciplinary meeting correspondence was considered the definitive record; 5) prevalence of “BE at the time of EA diagnosis” and “BE at the time of index EGD”. “BE at the time of EA diagnosis” includes all patients with known history of BE as well as those newly diagnosed with BE at the time of EA diagnosis. “BE at the time of index EGD” are cases with evidence of BE on index EGD, regardless of whether they had prior history of BE; 6) prognostic variables including treatment intent and 1-year survival rate; and 7) plans for surveillance and follow-up care.

### Root-cause analysis

**Fig. 2:**
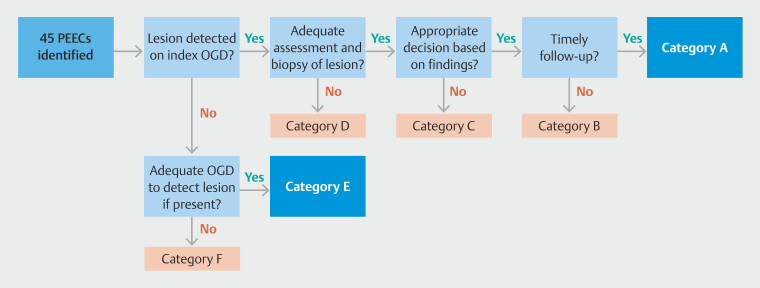
Flowchart delineating the root-cause analysis system.

A root-cause analysis system, adapted from Kamran et al 7 , was employed to divide the identified PEEC cases into six categories. This process involved the following sequential query response system ( Fig. 2 ). Judgements were made referencing the British Society of Gastroenterology (BSG), European Society of Gastrointestinal Endoscopy (ESGE), and the Australian Cancer Council (ACC) guidelines that were available to the endoscopists at the time the procedure was undertaken 19 20 21 .

1. Was the potentially premalignant lesion identified in the same segment as the subsequent PEEC? (yes — proceed to question 2; no — proceed to question 5).
2. Was the premalignant lesion adequately assessed? An adequate assessment required both: 1) adequate description and/or photo-documentation of the lesion (Prague criteria for BE, photograph or description of mucosal changes including nodules, esophagitis, or strictures); and 2) adequate biopsy of the lesion (Seattle protocol for BE). If the lesion was not assessed adequately due to factors such as poor tolerance or active esophagitis, an appropriate plan for early reassessment was needed. (yes — proceed to question 3; no —categorized as D).
3. Was the follow-up decision appropriate, according to accepted guidelines 19 20 21 ? Decision was assumed appropriate despite deviation from guidelines if patients opted for conservative management due to comorbidities or preference. (yes —proceed to question 4; no — categorized as C).
4. Was the decision followed in a timely manner? If the procedure was delayed without documentation of a cause outside our control (such as intercurrent illness or patient decision), they were assumed be administrative delays. Cases where follow-up EGDs were postponed due to factors outside our control were assumed to have been followed up in a timely manner. (yes — categorized as A; no — categorized as B.)
5. If the premalignant lesion was not identified, was the index EGD of adequate quality to detect the lesion if present? An adequate endoscopy required all of the following: 1) photo-documentation of J maneuver with adequate view quality; 2) photo-documentation of the esophagus with adequate view quality; and at least adequate patient tolerance. If the EGD report was unavailable, it was assumed the endoscopic examination was technically adequate and that there were no administrative delays. (yes — categorized as E; no — categorized as F).

Using this root-cause analysis system, PEECs were classified into six categories:

A: premalignant lesion detected, adequately assessed, appropriate follow-up decision and timely follow-up EGD performed.

B: premalignant lesion detected, adequately assessed and appropriate follow-up decision made, but follow-up EGD delayed due to administrative reasons.

C: premalignant lesion detected, adequately assessed, but follow-up decision inappropriate.

D: premalignant lesion detected, but assessment inadequate.

E: premalignant lesion not detected, but index EGD technically adequate to detect a lesion.

F: premalignant lesion not detected and index EGD inadequate to detect a lesion.

Categories B, C, D, and F were identified as potentially avoidable PEECs, including those delayed due to administrative factors and those attributable to endoscopist performance, encompassing situations of non-identification, inadequate assessment or biopsy of lesions, and improper follow-up plans.

### Data analysis

The proportion of patients who underwent EGD within 6 to 36 months prior to EA diagnosis was calculated from the entire cohort and stratified by various clinical, endoscopic and tumor characteristics including sex, ethnicity, indication for EGD, tumor site, stage of cancer, presence of BE at the time of EA diagnosis, location and acuity of EGD, treatment intent, and 1-year survival. The chi-squared test was used to compare differences across patient groups. *P* < 0.05 were considered statistically significant.

### Ethics

The study adhered to the national and regional data governance requirements. Engagements with Māori communities were facilitated through consultations with the Waitematā and Te Toka Tumai Auckland Māori Health Research Committee, which endorsed the project. The Health and Disability Ethics Committee provided ethical approval for the conduct of this study.

## Results

### Study subjects

**Fig. 3:**
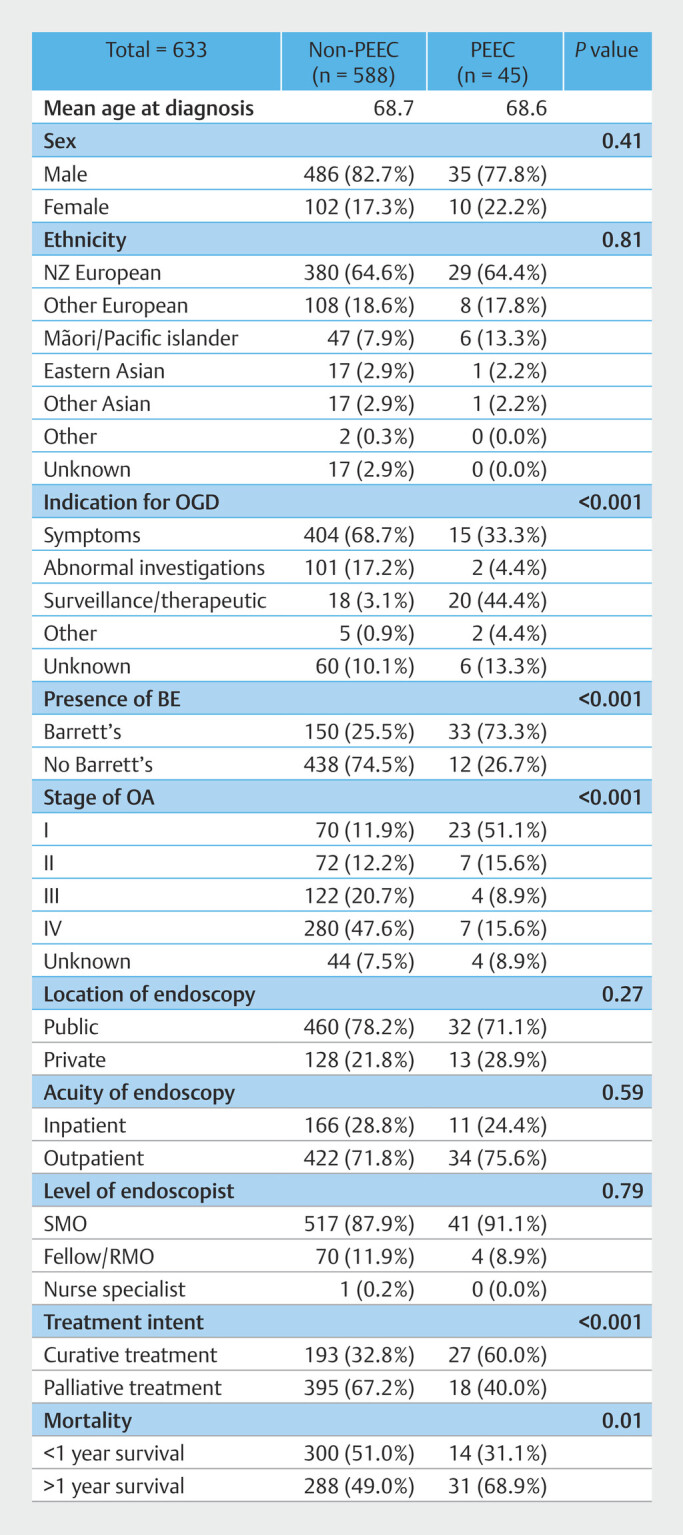
Characteristics of PEECs compared with non-PEECs. BE, Barrett’s esophagus; EA, esophageal adenocarcinoma; EGD, esophagogastroduodenoscopy; NZ, New Zealand; PEEC, post-endoscopy esophageal adenocarcinoma; RMO, resident medical officer; SMO, senior medical officer.

The study commenced with an initial cohort of 1,592 patients, all diagnosed with either esophageal or gastric cancers. After excluding 959 patients, 633 cases of EA satisfied the inclusion criteria ( Fig. 1 ). Of these, 45 (7.1%) were classified as PEEC. Patient demographic details, endoscopic findings, oncological characteristics, and treatment details for PEEC and non-PEEC groups are presented in Fig. 3 .

Among 633 patients diagnosed with EA, the majority (82.3%) were male, with a mean age of 68.7 years at diagnosis. A significant proportion was of European descent (82.9%) and 41.5% had a smoking history. Demographic characteristics were not significantly different between the groups.

### Cancer details

Among the 633 patients with EA, 571 (90.2%) had cancers in the lower esophagus or GOJ, whereas only 49 (7.7%) had cancers in the upper or mid-esophagus. Tumor locations did not differ significantly between the groups.

A total of 183 (28.9%) had BE at the time of EA diagnosis, either diagnosed previously or at the time of EA diagnosis. Patients in the PEEC group were more likely to have BE (73.3% vs. 25.5%). A total of 413 (65.2%) had advanced stage cancers (stage 3 or 4), whereas only 93 (11.2%) had stage 1 cancers. Stage 1 cancers were significantly more common in the PEEC group (51% vs 11.9%)

Curative treatments were received by 220 (33.2%) and 319 (50.4%) were alive 12 months after diagnosis. Curative treatment and 12-month survival rates were higher in the PEEC group (60.0% vs. 32.8% and 68.9% vs. 48.9%, respectively).

### Endoscopy details

Five hundred twenty-two (82.5%) of 633 endoscopies were conducted to investigate clinical symptoms, abnormal laboratory or imaging studies, whereas surveillance of established pathology accounted for 38 of the procedures (6.0%). A significantly higher proportion of patients in the PEEC group had their cancers diagnosed on surveillance EGD compared with those in the non-PEEC group (44.4% vs. 3.0%).

Four hundred ninety-two (77.7%) were identified within the public healthcare sector and 177 (27.9%) were detected through an inpatient EGD. Most procedures (558; 88.2%) were performed by senior medical officers and only a minority (26; 3.8%), were conducted concurrently with colonoscopies. Statistical analysis revealed no significant differences in these endoscopic factors between the groups.

### Root-cause analysis

**Fig. 4:**
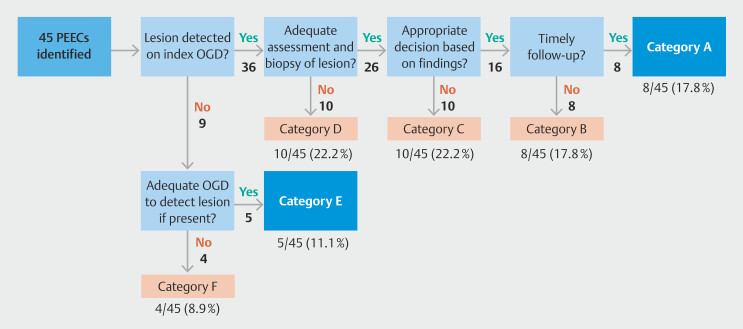
Flowchart delineating the root-cause analysis system and PEEC categorization.

In our cohort of 45 patients with PEEC, 36 (80%) had esophageal mucosal lesions found during their index EGD ( Fig. 4 ). Ten (27.8%) of these 36 patients had their lesions inadequately assessed: lesions were not adequately described nor photographed and/or sufficiently biopsied (categorized as D). BE was confirmed in 30 (83%) of these cases. Other mucosal lesions found on index EGDs included four cases described as “reflux esophagitis” and two cases described as “mucosal erythema” or “non-specific mucosal change”.

Among the 26 patients with properly assessed lesions, 10 patients had inappropriate follow-up decisions (categorized as C). This included two cases with HGD, seven with low-grade dysplasia, and one with indeterminate dysplasia. Of the remaining 16 patients, eight patients had their correctly planned EGDs delayed for administrative reasons (categorized as B); six of these delayed EGDs were early follow-up EGDs after an inadequate index EGDs due to factors such as presence of significant inflammation.

The remaining eight patients had adequate endoscopic assessment and appropriate follow-up decisions which were executed correctly (categorized as A). Patients in whom programmed EGDs were delayed for reasons outside our control were also included in this category; two patients had recurrent hospitalizations for intercurrent illnesses during follow-up period and one patient opted out of follow-up due comorbidities.

Of the nine index EGDs where no pathology was identified, five patients underwent technically sufficient EGDs capable of identifying an existing pathology (Category E), whereas four had technically deficient EGDs, potentially missing lesions (Category F).

**Fig. 5:**
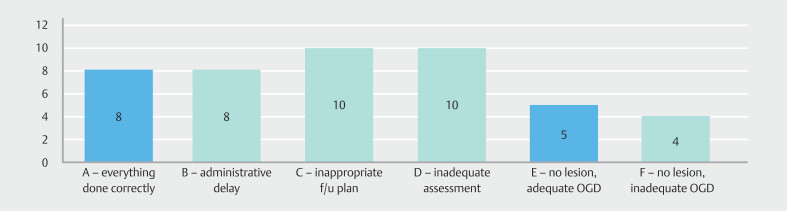
PEEC categorization according to root-cause analysis system. Potentially avoidable categories of PEEC are shown in red.

A significant proportion—32 of 45 PEEC cases (71.1%)—were categorized as B, C, D or F) and, therefore, classified as potentially avoidable ( Fig. 5 ).

### Barrett’s esophagus

Only 28.3% of the total study participants had BE at time of EA diagnosis (either newly diagnosed at time of EA diagnosis or had a previous documented history of BE). One contributing factor may be that endoscopy, or histology reports often did not comment on presence of Barrett’s mucosa when adenocarcinoma was identified—likely because the focus of documentation shifted to the malignancy, or because extensive tumor burden made assessment of underlying BE difficult. In addition, prior history of BE was inconsistently documented in the electronic medical records (EMRs), which may have led to underestimation. Patients in the PEEC group were more likely to have BE at time of EA diagnosis compared with the non-PEEC group (73.3% vs. 25.5%).

Thirty-four of 45 PEEC cases (75.6%) occurred in patients who had evidence of BE on their index EGD. Of these, 20 cancers (58.8%) were detected during surveillance endoscopies, 19 (55.9%) were stage 1 cancers, and 24 (70.6%) received treatment with curative intent.

**Fig. 6:**
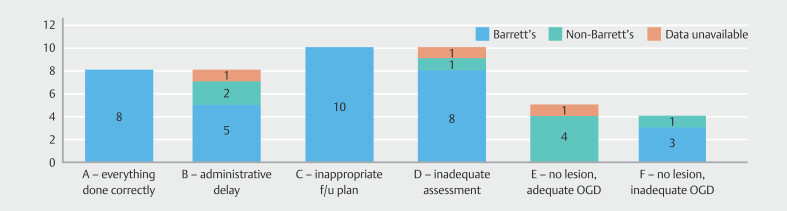
Patients with evidence of BE on index EGD in different PEEC categories.

Within the cohort of 32 potentially avoidable PEECs, 26 (81.2%) had evidence of BE on their index EGD ( Fig. 6 ); administrative delays accounted for five cases, 10 were due to inappropriate follow-up plans, eight resulted from inadequate endoscopies and/or biopsies, and three were due to failure to identify any lesion during an insufficient EGD.

## Discussion

This retrospective study is the first in New Zealand to investigate the rate of PEECs and conduct a root-cause analysis. We acknowledge that esophageal squamous cell carcinoma (SCC) is prevalent worldwide and that it may be over-represented in PEECs. However, we specifically focused on EAs because New Zealand has predominantly White population and adenocarcinomas are the predominant subtype of esophageal cancer in New Zealand. It also has a well-characterized premalignant condition—BE—which allows for structured surveillance and root-cause analysis, particularly for assessing missed opportunities in endoscopic evaluation.

The main strength of the study was our access to comprehensive EMRs, which facilitated acquisition of extensive data on factors that have been challenging to compile in prior research. This includes indications for endoscopy, stage at diagnosis, surveillance plans, reasons for deviations from surveillance plans, and history of BE.

In our study, 45 of 633 patients (7.1%) diagnosed with EA were classified as having PEECs. This prevalence aligns with rates reported in analogous international PEUGIC studies and implies that approximately 7.1% of EAs may be overlooked during an EGD, with our root-cause analysis attributing 71.1% of these cases to preventable factors. However, this statement presumes a relatively protracted natural progression of EA, assuming an arbitrary 3-year period for development of a new EA. It is conceivable that the natural history of some EAs is sufficiently rapid that a new EA can develop within 3 years. It is also possible that small early PEECs may be unavoidable despite thorough evaluation and adherence to guidelines. Under such circumstances, it is more judicious to postulate that only stage 2 to 4 cancers identified within 6 to 36 months following an EGD represent potentially missed diagnoses, equating to a more conservative PEEC incidence of 22 of 633 cases (3.5%). An alternative approach would be to adopt a 6- to 12-month interval as a cut-off, yielding an even lower PEEC rate of 14 of 633 cases (2.2%). Despite the scarcity of research on the natural progression of untreated EA, emerging evidence suggests a gradual advancement of dysplasia in patients with BE, with a prospective study from 2021 reporting an annual HGD or EA incidence of 0.78% among patients with BE 15 .

The comparative analysis between PEECs and non-PEECs revealed that in the PEEC group, a higher proportion of cases were diagnosed with stage 1 EAs (51.1% vs 11.9%), identified during surveillance EGDs (44.4% vs 3.1%), and had preexisting BE (73.3% vs 25.5%). Furthermore, these patients were more likely to receive curative treatments (60% vs 32.8%) and to surpass the 1-year survival threshold (68.9% vs 49%). These findings imply that some index EGDs were integrated within a planned BE surveillance program, allowing earlier cancer detection. In other words, at least some of these early cancers detected on surveillance may reflect a successful surveillance programmed rather than evidence of overlooked EA, even though they were categorized as PEECs according to our methodology.

Despite higher prevalence of PEECs among patients with BE, only 58.8% of the 34 PEECs with evidence of BE at the time of index EGD were identified through surveillance endoscopies. The remainder (41.2%) were diagnosed following the emergence of alarm symptoms or investigations. This may suggest potential for improvements in our BE surveillance program.

Failures to diagnose EA on EGD can result from several factors. Kamran et al. highlighted that the most prevalent causes for PEUGICs were suboptimal evaluation of premalignant conditions, insufficient endoscopic quality, and poor surveillance planning 7 . Our root-cause analysis found similar results with inappropriate follow-up plans (22.2%) and inadequate assessment/biopsy (31.1%) being the most frequent reasons, followed by administrative delays (17.8%). Notably, 75% of the PEECs attributed to administrative delays involved delayed early follow-up after inadequate assessments due to factors such as presence of inflammation. The guidelines from the BSG, ESGE, and ACC recommend repeat EGD 6 weeks after optimization of antireflux therapy for such patients, and yet, these repeat EGDs are frequently delayed for administrative factors 19 20 21 .

The BSG, ESGE, and ACC guidelines also specify surveillance intervals for BE patients, depending on length of BE and presence of dysplasia 19 20 21 . We applied these guidelines to assess appropriateness of endoscopist decisions regarding surveillance intervals. In cases in which the decisions did not comply with the guidelines, we still considered the decisions appropriate if patients opted for conservative management due to comorbidities or preferences. It is possible that some of these intentional deviations were not documented and not recognized by study investigators, resulting in overestimation of avoidable PEECs in category C.

The guidelines also outline specific quality standards for BE surveillance and biopsy 19 20 21 . Unfortunately, adherence to biopsy guidelines for BE surveillance has been variable in the past 22 . In this study, we found that 31.1% of PEECs occurred due to inadequate assessment/biopsy. In addition, only 25 of 45 (55.5%) index EGDs took more than three biopsies, despite the high prevalence of BE in patients with PEECs (75.6%), indicating inadequate adherence to the Seattle protocol. Furthermore, we were unable to determine whether some other quality standards were met during these EGDs; for example, information about inspection time was not available. We were also more lenient regarding photo-documentation than published guidelines because we only required one photograph of the premalignant lesion, one photograph of the esophagus in forward view, and one photograph of the GOJ in retroflexed position for the EGD to be technically adequate.

Another notable limitation of this study pertained to missing data, particularly regarding patients who underwent EGDs in private healthcare. In five instances in which EGD reports remained unattainable, certain assumptions were necessitated in the root-cause analysis, as detailed previously. Moreover, identification of patients who underwent their index EGD in the private sector was facilitated through our access to their histology reports. In cases in which no biopsies were taken, it was impossible to ascertain whether EGDs had been performed in the private sector. Consequently, there exists a possibility of slight underestimation in PEEC rates, because instances of private index EGDs in which no biopsies were taken might have been overlooked.

The study's demographic confinement to a single region predominantly inhabited by Europeans restricts its generalizability to a broader population. Moreover, the restricted sample size and limited number of PEECs identified precluded comprehensive subgroup analyses and pose challenges in drawing definitive conclusions. This also was a retrospective study, inherently susceptible to biases and confounding, thereby precluding establishment of causality. Furthermore, this study solely focused on EAs while excluding other types of UGICs, such as SCCs. Although our decision to concentrate exclusively on EAs allowed for a focused investigation of BE surveillance, generalizability of findings to other types of UGIC and comparison with international PEUGIC data are limited.

## Conclusions

In conclusion, this study provides valuable insights into PEECs in Auckland, showing a prevalence comparable to international PEUGIC studies. Root-cause analysis revealed that a significant proportion were potentially avoidable, with factors like suboptimal follow-up decisions, insufficient evaluation during endoscopic procedures, and administrative delays being the common contributors. Despite its limitations, the study highlighted shortcomings in adherence to BE surveillance guidelines and provides data to inform improvements in clinical practice. Initiatives aimed at enhancing guideline compliance, improving the quality of endoscopic evaluations, and curtailing administrative delays may help diminish PEEC incidence and ameliorate patient outcomes.
